# Future Orientation among Students Exposed to School Bullying and Cyberbullying Victimization

**DOI:** 10.3390/ijerph15040605

**Published:** 2018-03-27

**Authors:** Sara B. Låftman, Susanne Alm, Julia Sandahl, Bitte Modin

**Affiliations:** 1 Department of Public Health Sciences, Stockholm University, SE-106 91 Stockholm, Sweden; bitte.modin@su.se; 2 Swedish Institute for Social Research (SOFI), Stockholm University, SE-106 91 Stockholm, Sweden; susanne.alm@sofi.su.se; 3 Department of Criminology, Stockholm University, SE-106 91 Stockholm, Sweden; julia.sandahl@criminology.su.se

**Keywords:** future orientation, future expectations, bullying victimization, bullying perpetration, well-being, school

## Abstract

Future orientation can be defined as an individual’s thoughts, beliefs, plans, and hopes for the future. Earlier research has shown adolescents’ future orientation to predict outcomes later in life, which makes it relevant to analyze differences in future orientation among youth. The aim of the present study was to analyze if bullying victimization was associated with an increased likelihood of reporting a pessimistic future orientation among school youth. To be able to distinguish between victims and bully-victims (i.e., students who are both bullies and victims), we also took perpetration into account. The data were derived from the Stockholm School Survey performed in 2016 among ninth grade students (ages 15–16 years) (*n =* 5144). Future orientation and involvement in school bullying and in cyberbullying were based on self-reports. The statistical method used was binary logistic regression. The results demonstrated that victims and bully-victims of school bullying and of cyberbullying were more likely to report a pessimistic future orientation compared with students not involved in bullying. These associations were shown also when involvement in school bullying and cyberbullying were mutually adjusted. The findings underline the importance of anti-bullying measures that target both school bullying and cyberbullying.

## 1. Introduction

Future orientation can be defined as an individual’s thoughts, beliefs, plans, and hopes for the future [1]. While people formulate beliefs and hopes for the future across the entire life course, thoughts and plans about one’s future are of special relevance during adolescence and young adulthood, as this is a period of life when people tend to choose a direction for their education, occupation, and other central aspects of life [2,3]. Future orientation can be separated into three components: a cognitive, a motivational, and an affective one. The affective component, referring to the degree of optimism or pessimism towards one’s future, is the most commonly studied aspect of future orientation [4]. The affective component of future orientation is also the one focused upon in the present study. 

Previous studies have indicated that future orientation is an independent predictor of outcomes later in life. For instance, adjusting for household socioeconomic status in childhood, adolescents’ future orientation, reported at the age of 12–13 years, has been shown to predict both labor market attachment and premature death in adulthood [5,6]. An earlier study has also shown that a positive future orientation protects against feelings of hopelessness and depressive symptoms among adolescents exposed to familial and peer emotional victimization [7], indicating that an optimistic future orientation may act as a buffering resource. Less is however known about the direct association between bullying victimization and future orientation. The overall aim of the present study is to assess whether bullying victimization is associated with a greater likelihood of reporting a pessimistic future orientation, using large-scale survey data collected in 2016 among ninth grade students (ages 15–16 years) in Stockholm, Sweden. 

Bullying is commonly defined as repeated negative actions performed by one or more other persons and directed towards an individual [8]. Such negative actions may be direct, either physical (e.g., hitting, kicking) or verbal (teasing, calling names), and they may be indirect (e.g., ostracism). Beside the element of repetition, central features of bullying include the existence of an actual or a perceived power imbalance between the perpetrator(s) and the victim, and that the negative actions are intended to do harm [9]. In an international perspective, Sweden has low rates of bullying. For instance, in the international Health Behaviour of School-aged Children (HBSC) study of 2013/14, nine percent of ninth grade students in Sweden reported to have been bullied at school at least once during the past couple of months (HBSC average: 23 percent). With regards to cyberbullying, in the same survey, six percent of Swedish ninth grade students reported to have been cyberbullied by messages at least once (HBSC average: 11 percent) and six percent to have been cyberbullied by pictures at least once (HBSC average: nine percent) [10]. 

According to symbolic interactionism [11], an individual’s perception of his or her “self” is constructed and shaped in the social interaction with others. As a result of the repeated exposure to negative actions by the part of others, bullied students may perceive themselves in negative light [12,13]. Feelings of shame indicate that an individual does not fulfil the group standards [14]. Given the significant role of group membership for adolescents’ self-esteem and identity formation [15], being excluded from the peer group may invoke feelings not only of shame, but also of low self-worth and depressive feelings. Indeed, a significant amount of research has shown that being bullied is positively associated with psychosomatic problems [16], but also with lower well-being in terms of poorer self-esteem [17] and lower health-related quality of life [18]. It seems reasonable to assume that the experience of being bullied may be reflected also through a pessimistic view of one’s future, although empirical knowledge about this association is lacking. Some previous studies have reported an association between a pessimistic future orientation and involvement in bullying perpetration [19,20,21]. The association was interpreted in light of other studies that reported links between a pessimistic future orientation and a greater likelihood of engaging in problem behaviors [1,4]. Thus, in these studies, low future orientation was investigated as a dependent, rather than an independent factor. While both directions of the relationship between future orientation and bullying experience are reasonable when focusing on perpetration, concerning bullying victimization, it is most logical to consider future orientation as an outcome. 

However, from previous research we know that not only the victims of bullying, but also the perpetrators, report more psychosomatic problems than those not involved in bullying [16]. In addition, there also exists some overlapping between the groups, in that some individuals are both victims and perpetrators [16]. This category of students, often labelled “bully-victims”, tend to report the worst mental health [12,22]. Accordingly, when analyzing bullying victimization in relation to various types of outcomes, it is an advantage to also take perpetration into account, since distinguishing between students who are only victims and those who are bully-victims entails a more fine-grained picture. It is a strength of the current study that we were able to do this. 

Furthermore, some empirical studies distinguish between school bullying and cyberbullying. The former concept covers types of bullying that are performed in the school setting, whereas cyberbullying is conceptualized as being performed electronically through computers and mobile phones. The two forms of bullying have many features in common, but there are also differences. School bullying is most often performed face-to-face in the school setting. In contrast, cyberbullying is not restricted by time and place, but can occur anytime and anywhere. In addition, due to its digital nature, acts of cyberbullying can easily be repeated and also witnessed by a wider audience [23]. While there is an overlap in students’ involvement in school bullying and cyberbullying [23,24,25,26,27,28], studies have shown that involvement in these two forms of bullying have independent negative associations with students’ psychological health [23,26,29,30,31]. Accordingly, we hypothesize that victimization of school bullying and of cyberbullying are independently associated with students’ future orientation, too. 

The aim of this study was to analyze the association between bullying victimization and future orientation among students, whilst also taking into account perpetration. We studied victimization of both school bullying and of cyberbullying. Our hypotheses were:

**Hypothesis** **1** **(H1).** Victimization of school bullying is associated with an increased likelihood of reporting a pessimistic future orientation, also when adjusting for perpetration of school bullying.

**Hypothesis** **2** **(H2).** Victimization of cyberbullying is associated with an increased likelihood of reporting a pessimistic future orientation, also when adjusting for perpetration of cyberbullying.

**Hypothesis** **3** **(H3).** The associations postulated in H1 and H2 exist independently of each other.

## 2. Methods 

### 2.1. Data Material

The Stockholm School Survey (SSS) is a repeated cross-sectional survey performed every two years among students in the ninth grade of elementary school (ages 15–16 years) and in the second grade of upper secondary school (ages 17–18 years) in all public schools and a number of independent schools in Stockholm municipality. Students complete the questionnaires by paper and pencil in the classroom. The survey includes questions on risk behaviors such as alcohol use, drug use, smoking and delinquent behavior, but also covers areas such as bullying, school climate, and health [12,23,32,33,34,35,36,37]. The 2016 survey also included a question on students’ future orientation. In the present study, we analyzed this newly collected data among students in the ninth grade of elementary school. The response rate was 78 percent [32], resulting in a total of 6465 participating students. Observations with internal non-response on any of the variables used in the analyses were omitted, i.e., listwise deletion (*n =* 1321), leading to a study sample of 5144 students.

Since the Stockholm School Survey is completed anonymously and the students do not provide any information on personal identification, the Regional Ethical Review Board of Stockholm has declared that the data are not subject to consideration for ethical approval (2010/241-31/5).

#### Measures

Future orientation was captured by the question, “If you compare your future prospects to those of most others of your age, do you believe that yours are worse, just as good, or better?” with five response categories: (1) “Much worse”; (2) “Slightly worse”; (3) “Just as good”; (4) “Slightly better” and (5) “Much better”. The first two response categories were classified as “Pessimistic” and contrasted against the three latter which were classified as “Not pessimistic”. The measure has been used previously [6,38]. 

School bullying was constructed from two questions, one about victimization and one about perpetration. School bullying victimization was assessed from the question: “During the past school year, how often have you been bullied or harassed at school?” with the response categories (1) “I have not been bullied”; (2) “It has happened occasionally”; (3) “2 or 3 times a month”; (4) “About once a week” and (5) “Several times a week”. Being a victim of school bullying was defined as having been bullied or harassed two to three times a month or more often. School bullying perpetration was captured by the question: “Have you taken part in the bullying or harassment of other students this school year?” with the same response categories as for victimization, as well as the category “Don’t know”. Being a perpetrator of school bullying was defined as having bullied other students two to three times a month or more often. Students who responded “Don’t know” were coded as missing (*n =* 149). Based on these two measures, four categories of school bullying were created: not involved, victim only, bully only, and bully-victim. The items have been used in previous studies [12,33,35,36]. These questions were asked after the opening question, “Have you felt bullied or harassed at school this school year?”, with the response option, “No”, and a list of non-mutually exclusive items on different types of victimization: “I have been mocked, ridiculed, called names or been teased in an unpleasant and hurtful way”; “I have been frozen out by other students”; “I have been hit, kicked, pushed or locked in”; “Another student has spread lies or false rumors about me and tried to get others to dislike me”; “Someone has taken money or something else from me, or other things of mine have been ruined”; “I have been threatened or forced to do things I didn’t want to do”; “Teachers have psyched me out or been mean in another way to me” and “I have been bullied in another way”. Students who had only ticked that they were bullied by a teacher, and none of the other statements, were not classified as being bullied in the present study. 

Cyberbullying was constructed from two questions which were posed immediately after the questions on school bullying, one about victimization and one about perpetration. Cyberbullying victimization was measured through the question “Have you been bullied or harassed on the Internet or by text messaging (SMS/MMS) this school year?” The response categories were “Yes”, “No”, and “Don’t know” where the last category was coded as missing (*n =* 338). Cyberbullying perpetration was assessed through the question “Have you taken part in the bullying or harassment of other students on the Internet or by text messaging this school year?” Also for this question, the response categories were “Yes”, “No”, and, “Don’t know”, with the last category coded as missing (*n =* 218). Based on these two measures, four categories of cyberbullying were created: not involved, victim only, bully only, and bully-victim. The items have been used in previous studies [23,36,37]. 

A set of socio-demographic characteristics were included as control variables.

Gender was measured by the question “Are you a boy or a girl?” The response categories were “Boy” and “Girl”. 

Family structure was measured through the question, “Which people do you live with?” and a list of boxes to be marked. Those who marked “Mother” and “Father” were categorized as living with two parents in one household and were contrasted against all others. 

Parental university education was captured by the question “What is the highest education your parents have?” The response categories (to be marked for mother and father separately) were: “Old elementary school (folkskola) or elementary school (max nine years schooling)”, “Upper secondary school”, “University and university college”, and “Don’t know”. Students who marked “University and university college” for one or both parents were classified as having at least one parent with university education, and contrasted against all others. Although reliability in adolescents’ reports on their parents’ education is known to be limited, and with relatively high levels of non-response [39,40], this variable was used as a proxy for parental university education. 

Migration background was assessed by the question “How long have you lived in Sweden?” The response categories were “All my life”, “10 years or more”, “5–9 years” and “Less than five years”. The variable was dichotomized to capture students who had lived in Sweden at least 10 years (i.e., approximately during the elementary school years) vs. those who had lived in Sweden less than 10 years. 

### 2.2. Statistical Method

Since the outcome was dichotomous, binary logistic regression analysis was applied, using the “logistic” command in Stata 15. To adjust for the fact that students were nested in schools, robust standard errors clustering at the school level were estimated using Stata’s “robust cluster” command. The 5144 students in the study sample were distributed across 125 schools. 

## 3. Results 

Descriptives of the data are presented in Table 1. According to our operationalization, 17.0% of the students reported to have a pessimistic future orientation. Relatively small shares of students were involved in school bullying: 4.1% reported to be victims of school bullying, 1.7% to be bullies, and 0.5% to be both bullies and victims. Cyberbullying was more common, which is most likely due to the fact that the measures of cyberbullying do not take frequency into consideration—only whether or not it has occurred during the past school year. In all, 8.1% of the students reported to be victims of cyberbullying, 2.0% to be bullies, and 2.2% to be both bullies and victims of cyberbullying. In the Appendix A, Table A1, descriptive statistics are presented for the cases that were excluded due to listwise deletion. 

Table 2 presents the correspondence between students’ involvement in school bullying and cyberbullying, respectively. The percentages show that there was a clear, but far from perfect, overlap between involvement in school bullying and cyberbullying. 

Table 3 demonstrates the distribution of students reporting a pessimistic future orientation by involvement in school bullying and in cyberbullying, respectively. For both forms of bullying, having a pessimistic view of one’s future was more common among victims and bully-victims, but also among perpetrators, compared with students who were not involved. For both forms of bullying, the largest proportions of students with a pessimistic future orientation were found in the bully-victim category, followed by the victim only category. 

Results from binary logistic regression analyses are presented in Table 4. The estimates shown are odds ratios which demonstrate the likelihood of reporting a pessimistic future orientation. The column labeled “Crude” presents analyses that include one independent variable at a time, adjusted only for gender, while Models 1–3 adjust simultaneously for bullying and the control variables. The results from the crude analyses showed that, adjusting only for gender, victims and bully-victims of both school bullying and of cyberbullying had a higher likelihood of reporting a pessimistic future orientation compared with those who were not involved in bullying at all. For both types of bullying, the estimates were strongest for the bully-victim categories. In addition, students who were only bullies also displayed an increased likelihood of reporting a pessimistic future orientation. Model 1 includes school bullying and the full set of control variables. Compared to the results from the crude analysis, the estimates of victims only and of bully-victims were slightly altered but remained statistically significant, whereas the estimate of bullies only turned non-significant. Model 2 includes cyberbullying and the full set of control variables. As an analogue to school bullying, being a victim only or a bully-victim of cyberbullying remained substantially and significantly linked to an increased likelihood of reporting a pessimistic future orientation, while the estimate of being a bully only was no longer statistically significant. In Model 3, mutually adjusting for school bullying and cyberbullying as well as the full set of control variables, all estimates of the bullying categories were somewhat attenuated. Yet, for victims and bully-victims of school bullying and of cyberbullying, the associations with a pessimistic future orientation remained substantial and statistically significant. However, for students classified as “bullies only”, the associations with future orientation were not statistically significant—neither for school bullying nor cyberbullying.

## 4. Discussion

This study hypothesized that victimization of school bullying and of cyberbullying was associated with an increased likelihood of reporting a pessimistic future orientation. It also hypothesized that victimization of school bullying and of cyberbullying demonstrate independent associations with a pessimistic future orientation. Using recent survey data collected among students in Stockholm, we showed that both victims and bully-victims of school bullying and of cyberbullying were more likely to report a pessimistic future orientation than those who were not involved in bullying. The finding was expected. Being bullied is a manifestation of the group’s disapproval of an individual, which can evoke feelings of shame as well as a negative self-perception. Earlier studies have demonstrated that victimization is linked to more psychosomatic problems [16], but also to poorer self-esteem [17] and lower health-related quality of life [18], and it seems reasonable to assume that feelings of shame and a negative self-perception can imply also a pessimistic view of one’s own future. This study is, to the best of our knowledge, among the first to show that victimization is associated with a pessimistic future orientation.

In support of earlier studies of the associations between different forms of bullying and psychological health [23,26,29,30,31], we found that being a victim or a bully-victim of school bullying or of cyberbullying demonstrated independent associations with the likelihood of reporting a pessimistic future orientation. This may partly have to do with the fact that although involvement in school bullying and in cyberbullying overlap, it is not the exact same individuals who are involved in the two forms of bullying in the same ways. Another, non-mutually exclusive, possible explanation is that since both school bullying and cyberbullying have unique features, they may also have independent effects on students’ future orientation. 

Some earlier studies have reported a link between a pessimistic future orientation and perpetration of bullying [19,20,21]. In this study, we did not find that students who were “bullies only” of school bullying or of cyberbullying were more likely to report a pessimistic future orientation compared with those who were not involved. One possible explanation of this discrepancy of results is that we took into account both victimization and perpetration in the analyses, investigating categories of involvement in bullying and their associations with future orientation. 

In light of earlier longitudinal studies which have shown that adolescents’ future orientation, reported at the age of 12–13 years, predicts their actual future in terms of labor market outcomes in adulthood but also premature death, even when adjusting for socioeconomic background in childhood [5,6], the findings of the present study are important. Accordingly, the result that being a victim or a bully-victim of school bullying or of cyberbullying was linked with a greater likelihood of a pessimistic future orientation, which in turn could have negative effects on outcomes later in life [5,6], emphasizes the importance of efficient anti-bullying measures. 

While the present study has several merits, most notably the large-scale data material which made it possible to study victimization of both school bullying and of cyberbullying and their links to students’ future orientation, there are also limitations. Since the data were cross-sectional we were not able to investigate the direction of the causality between bullying victimization and future orientation. While it seems intuitively plausible that bullying victimization can cause a pessimistic future orientation, this may not necessarily be the case for perpetration. Yet, given the certain overlap between victimization and perpetration, we included both aspects to be able to refine the different categories of involvement in bullying. To empirically disentangle the causal relationships between bullying victimization and future orientation would however require longitudinal data. Furthermore, we cannot rule out the possibility of confounding, i.e., that there were characteristics or experiences among students which affected both the risk of bullying victimization and their future orientation. Another limitation concerns the rather high internal non-response, as well as the related fact that a number of students who answered “Don’t know” to the questions on school bullying perpetration and cyberbullying victimization and perpetration were excluded from the analyses. Furthermore, the measures have not been formally validated. Yet, the shares of students involved in school bullying and in cyberbullying reported here do not deviate substantially from those reported in other investigations of the same age group. In the Swedish part of the Health Behaviour of School-aged Children (HBSC) study of 2013/14, about three percent of ninth grade students reported to have been bullied at least 2–3 times a month during the past months, and about 2 per cent to have bullied others [41]. In the same survey, about six percent of ninth grade students reported to have been cyberbullied by messages and about six percent to have been cyberbullied by pictures at least once during the past months [10,41] (there was no question on cyberbullying perpetration). Furthermore, the descriptives of the study sample and of the students that were excluded due to listwise deletion, respectively, indicated that overall the attrition was not heavily skewed in relation to the variables under study, although it should be acknowledged that in particular perpetrators of bullying were somewhat underrepresented in the study sample. Finally, the analyses were based on data collected among students in Stockholm, implying that the scope of the generalizability to other contexts is limited. While studies of cross-national data have demonstrated that school bullying and cyberbullying are less common in Sweden than in other countries [10], future research should investigate whether victimization of school bullying and of cyberbullying is linked with a more pessimistic future orientation also in countries with higher rates of bullying. An implication of the relatively low prevalence of bullying in Sweden is also that, despite our large-scale data, some of the categories under study contained rather small numbers of individuals. Thus, in particular the results regarding the school bullying category “bully-victim” should be interpreted with some caution. The relatively small numbers of students involved in bullying also refrained us from analyzing how different combinations of involvement in school bullying and in cyberbullying were associated with future orientation. Assessing the interactive effects of different forms of bullying on students’ future orientation is a relevant task for future research. 

## 5. Conclusions

The present study demonstrated that being a victim or a bully-victim of school bullying or of cyberbullying was linked with an increased likelihood of a pessimistic future orientation. The results reflect and extend earlier research which has shown that victimization is associated with more psychosomatic problems [16], lower self-esteem [17] and poorer health-related quality of life [18], and that involvement in school bullying and in cyberbullying have independent effects on student health outcomes [23,26,29,30,31]. To conclude, the present study contributes to the literature by showing an association between victimization and a pessimistic future orientation. Not least since earlier longitudinal studies have demonstrated direct associations between a pessimistic future orientation in adolescence and adverse outcomes later in life [5,6], the findings of this study underline the importance of effective anti-bullying measures that target both traditional school bullying and cyberbullying.

## Figures and Tables

**Table 1:** Descriptives, *n =* 5144.

	All (*n =* 5144)		Boys (*n =* 2546)		Girls (*n =* 2598)	
Variables	*n*	%	*n*	%	*N*	%
Future orientation						
Not pessimistic	4270	83.0	2105	82.7	2165	83.3
Pessimistic	874	17.0	441	17.3	433	16.7
School bullying						
Not involved	4819	93.7	2386	93.7	2433	93.6
Victim only	210	4.1	72	2.8	138	5.3
Bully only	86	1.7	64	2.5	22	0.9
Bully-victim	29	0.5	24	0.9	5	0.2
Cyberbullying						
Not involved	4513	87.7	2256	88.6	2257	86.9
Victim only	415	8.1	143	5.6	272	10.5
Bully only	104	2.0	76	3.0	28	1.1
Bully-victim	112	2.2	71	2.8	41	1.6
Family structure						
Two parents in the same household	3526	68.6	1779	69.9	1747	67.2
Other	1618	31.4	767	30.1	851	32.8
Parental university education						
No parent	1810	35.2	943	37.0	867	33.4
At least one parent	3334	64.8	1603	63.0	1731	66.6
Migration background						
≥10 years in Sweden	4626	89.9	2276	89.4	2350	90.4
<10 years in Sweden	518	10.1	270	10.6	248	9.6

**Table 2:** Involvement in school bullying by involvement in cyberbullying. Row percent (*n* within brackets). *n =* 5144.

	Cyberbullying			
School Bullying	Not Involved	Victim Only	Bully Only	Bully-Victim
Not involved	89.6 (4316)	7.1 (340)	1.6 (78)	1.7 (85)
Victim only	65.7 (138)	31.0 (65)	0.5 (1)	2.8 (6)
Bully only	52.3 (45)	9.3 (8)	25.6 (22)	12.8 (11)
Bully-victim	48.3 (14)	6.9 (2)	10.3 (3)	34.5 (10)

**Table 3:** Distribution of future orientation, by involvement in bullying. *n =* 5144.

	Not Pessimistic	Pessimistic	Significant Difference
**School bullying**			
Not involved (ref.)	83.9	16.1	-
Victim only	69.5	30.5	***
Bully only	74.4	25.6	*
Bully-victim	55.2	44.8	***
**Cyberbullying**			
Not involved (ref.)	84.2	15.8	-
Victim only	75.2	24.8	***
Bully only	76.0	24.0	*
Bully-victim	70.5	29.5	***

*** *p* < 0.001; * *p* < 0.05.

**Table 4:** Pessimistic future orientation by school bullying and cyberbullying status (reference category=not pessimistic future orientation). Odds ratios from binary logistic regressions. *n =* 5144.

	Crude		Model 1		Model 2		Model 3	
	OR	95% CI	OR	95% CI	OR	95% CI	OR	95% CI
**School bullying**								
Not involved (ref.)	1.00	-	1.00	-			1.00	-
Victim only	2.30 ***	1.71–3.11	2.33 ***	1.72–3.16			2.08 ***	1.50–2.89
Bully only	1.77 *	1.08–2.92	1.59	0.96–2.64			1.36	0.80–2.31
Bully-victim	4.18 ***	2.00–8.73	3.99 ***	1.84–8.64			3.19**	1.46–6.96
**Cyberbullying**								
Not involved (ref.)	1.00	-			1.00	-	1.00	-
Victim only	1.77 ***	1.37–2.31			1.76 ***	1.35–2.30	1.59 **	1.20–2.10
Bully only	1.67 *	1.09–2.55			1.46	0.95–2.25	1.35	0.86–2.11
Bully-victim	2.21 ***	1.51–3.23			2.14 ***	1.46–3.14	1.82 **	1.23–2.70
**Gender**								
Boy (ref.)	1.00	-	1.00	-	1.00	-	1.00	-
Girl	0.95	0.82–1.11	0.97	0.83–1.13	0.96	0.82–1.11	0.95	0.82–1.11
**Family structure**								
Two parents in the same household (ref.)	1.00	-	1.00	-	1.00	-	1.00	-
Other	1.52 ***	1.29–1.79	1.39 ***	1.18–1.65	1.39 ***	1.18–1.65	1.38 ***	1.17–1.64
**Parental university education**								
No parent (ref.)	1.00	-	1.00	-	1.00	-	1.00	-
At least one parent	0.53 ***	0.45–0.61	0.55 ***	0.47–0.64	0.55 ***	0.47–0.64	0.55 ***	0.47–0.64
**Migration background**								
≥10 years in Sweden (ref.)	1.00	-	1.00	-	1.00	-	1.00	-
<10 years in Sweden	1.21	0.91–1.59	1.05	0.80–1.38	1.04	0.80–1.37	1.03	0.79–1.36

*** *p* < 0.001; ** *p* < 0.01; * *p* < 0.05.

## Appendix A

**Table A1:** Descriptives of the cases excluded due to listwise deletion (*n* = 1321).

	Cases Excluded Due to Listwise Deletion	
Variables	*n*	%
Future orientation		
Not pessimistic	822	77.8
Pessimistic	234	22.2
*Missing*	*265*	
School bullying		
Not involved	830	91.3
Victim only	41	4.5
Bully only	32	3.5
Bully-victim	6	0.7
*Missing*	*412*	
Cyberbullying		
Not involved	535	86.2
Victim only	43	6.9
Bully only	26	4.2
Bully-victim	17	2.7
*Missing*	*700*	
Family structure		
Two parents in the same household	809	61.2
Other	512	38.8
*Missing*	*0*	
Parental university education		
No parent	527	39.9
At least one parent	794	60.1
*Missing*	*0*	
Migration background		
≥10 years in Sweden	1132	87.9
<10 years in Sweden	156	12.1
*Missing*	*33*	
Sex		
Boys	569	52.8
Girls	509	47.2
*Missing*	*243*	
